# Screening and evaluation of cytotoxicity and antiviral effects of secondary metabolites from water extracts of *Bersama abyssinica* against SARS-CoV-2 Delta

**DOI:** 10.1186/s12906-022-03754-3

**Published:** 2022-10-26

**Authors:** Never Zekeya, Bertha Mamiro, Humphrey Ndossi, Rehema Chande Mallya, Mhuji Kilonzo, Alex Kisingo, Mkumbukwa Mtambo, Jafari Kideghesho, Jaffu Chilongola

**Affiliations:** 1 Department of Wildlife Management, College of African Wildlife Management, CAWM, P.O. Box 3031, Mweka, Moshi, Kilimanjaro Tanzania; 2 Tanzania Industrial Research and Development Organization (TIRDO), P.O. Box 23235, Msasani, Dar es Salaam, Tanzania; 3 School of Pharmacy and Pharmacognosy, Muhimbili University of Health and Allied Sciences, P.O. Box 65014, Dar es salaam, Tanzania; 4 University of Dodoma, Dodoma, Tanzania; 5 Kilimanjaro Christian Medical University College, P.O. Box 2240, Moshi, Kilimanjaro Tanzania

**Keywords:** *Bersama abyssinica*, Bioactive compounds, Coviba Dawa, COVID-19, Traditional medicine, Tanzania

## Abstract

**Background:**

*Bersama abyssinica* is a common herb in Africa, with diverse medical uses in different areas. The plant is well-known in Tanzania for treating respiratory disorders such as TB, tonsillitis, bronchitis, and asthma, and it has lately been utilized to treat COVID-19 symptoms. Water extract of leaf and stem bark has been registered as an herbal medication known as 'Coviba Dawa' in Tanzania for the relief of bacterial respiratory infections. The extracts, however, have not been scientifically tested for their anti-viral activities. The aim of this work was to test for the cytotoxicity and antiviral effects of bioactive ingredients from *B. abyssinica* extracts against the Delta variant of the SARS-CoV-2 coronavirus.

**Methods:**

*B. abyssinica* leaves and stem bark were dried under shade in room temperature and then pulverized to obtain small pieces before soaking into different solvents. One hundred grams of each, leaves and stem bark, were extracted in petroleum ether, dichloromethane, ethyl acetate and methanol. Water extract was obtained by decoction of stem bark and leaves into water. Phenols, flavonoids, tannins, and antioxidants were confirmed as components of the extracts. Analysis of polar extracts of bark stem bark and leaves was done. Antiviral screening and cytotoxicity experiments were conducted in a Biosafety Level 3 (BSL-3) Laboratory facility according to International Standard Operating Procedures (SOPs).

**Results:**

By the use of LC–MS/MS analysis, this study confirmed the existence of four phenolic compounds in *B. abyssinica* water extract; 2,4-di-tert-butylphenol, 4-formyl-2-methoxyphenyl propionate, 7,8-Dihydroxy-4-methylcoumarin, and 2,3, 6-trimethoxyflavone with antioxidant activity. This study showed that, while the water extracts of *B. abyssinica* had significant antiviral activity against SARS Cov2 virus, it showed no cytotoxicity effect on Vero E6 cells. In particular, the water extract (Coviba dawa) showed 75% while ethylacetate fraction of *B. abyssinica* leaves showed a 50% in vitro viral inhibition, indicating that these substances may be useful for the development of future anti-viral agents.

**Conclusion:**

We therefore recommend isolation of compounds for further profiling and development with a broader concentration range. We further recommend studies that determine the antiviral activity of extracts of *B.abyssinica* on other viral pathogens of clinical concern.

**Supplementary Information:**

The online version contains supplementary material available at 10.1186/s12906-022-03754-3.

## Introduction

Coronavirus (SARS-CoV-2) causes Coronavirus disease (COVID-19)*,* a serious viral infectious disease of global concern [1]. This disease has greatly impacted global economy, mobility, socio-economy, and health systems [2, 3].

Despite major advances in epidemic preparedness, Africa remains uniquely susceptible to COVID-19 [4]. According to the Infectious Disease Vulnerability Index, 22 out of the 25 African countries are most susceptible to an infectious disease outbreak [4]. This unique vulnerability to Africa needs local creative solutions to control infectious diseases. For example, while lock-down was one of the strategies adopted by most European and American countries, and least applicable in Africa due to several reasons including unavailability of basic services to support ‘Lock Downs’. In addition to allopathic treatment, the use of traditional medicines was one of the most popular strategies adopted in African countries to relieve severe symptoms of COVID-19.

According to recent investigations, traditional remedies have the ability to relieve COVID-19 symptoms and perhaps cure the disease [5]. India, China, and Nepal have reported to produce effective compounds derived from medicinal plants to cure a wide range of viral diseases, including SARS-CoV-2 infection [6–9]. Medicinal plants have been shown to be potential to prevent symptoms related to COVID-19 though more pharmacological studies are required to prove their activities [10, 11]. A number of approaches varying from social to biological have been adopted to combat COVID-19 in Sub Saharan Africa [12]. One of these promising approaches has been the use of medicinal plants and spice mixtures with unknown active components to alleviate the severe symptoms usually associated with COVID-19 [13].

A wide range of active compounds that treat microbial disorders, including viral infections have been discovered in variety of medicinal plants including *Bersama abyssinica* [14, 15]. Herbal extracts with antiviral activity have been identified throughout West Africa, particularly in Benin and the Ivory Coast [16]. Despite the wide use of natural medicinal herbs to treat a wide range of diseases in Africa, only a few scientific studies have objectively established and validated the effectiveness of the active ingredients in these plants against the broad spectrum of infectious agents endemic to Africa [17].

*Bersama abyssinica* is among the well-studied plants in Africa, with diverse antimicrobial activities against bacteria and viruses [12, 13]. Recent study by Sinan et al. 2020 has revealed *B. abyssinica* to possess several active secondary metabolites with antioxidant, anti-respiratory and antimicrobial activities [18]. A previous study on local knowledge from southern Tanzania on the herbs for medicinal purposes found widespread usage of *B. abyssinica* for COVID-19 treatment [12]. Many other investigations have shown that the claimed bioactivity of phenolic compounds and gallic from *B. abyssinica* against viral infections, including COVID-19, is due to interference with viral RNA transcription and protein biosynthesis processes [19–21]. Despite the reported effectiveness and use of *B. abyssinica* against abroad range of pathogenic microbes, there is an apparent paucity of data specifically on its cytotoxic and antiviral effects against COVID-19. Therefore, this study was designed to evaluate the phytochemical, *in-vitro* cytotoxicity of *B. abyssinica* stem and leaf extracts on host cells and their antiviral activity against -SARS- CoV-2 virus.

## Material and methods

All methods were carried in accordance with relevant guidelines and regulations.

### Study site and collection of plant materials

*Bersama abyssinica* plant materials were obtained during the dry season of 2021 in the Isongole area, at a river line forest patch in the Rungwe District, Mbeya region (9^o^34′60.9 s 33^o^62′84 e). *B. abyssinica* plant materials were collected through collection permit # FMM 4052 and the plant was identified by Dr. Ester Mvungi from the University of Dar es salaam with the Voucher specimen number: ND.Zekeya Nos.01 which was deposited in the herbarium at the University of Dar es salaam. According to national (Tanzania) and international (IUCN) regulations and standards, the plant is of Least conservation concern. However, only aerial parts; leaves and stem bark were collected and dried in the shade at room temperature before being crushed into small pieces and soaked in various solvents. Extraction and phytochemical analysis were carried out at the Institute of Traditional Medicine of Muhimbili Institute of Health and Allied Sciences and the Government Chemist Laboratory Authority. Cytotoxicity and antiviral assays of the plant extracts were conducted at Basel University, Switzerland.

### Extraction chemicals and materials

Absolute Methanol (Fluka Chemie GmbH, Zwijndrecht, NL), Dimethyl sulfoxide (DMSO)(RFCL Limited, Hayana, India), Dichololomethane, ethyl acetate and Methanol (Loba Chemie Pvt Ltd, Mumbai, India), Ferric Chloride (FeCl_3)_, Ammonium hydroxide (NH_4_OH), Sulphuric acid (H_2_SO_4_) and 2–2-Diphenyl-1-picrylhydrazyl (DPPH) were used as extraction chemicals and for the analysis.

### Preparation of plant materials and extraction

The plant components were chosen based on their recognized efficacy as components of the Coviba Dawa®, a herbal preparation registered in Tanzania. Furthermore, for conservation purposes, no roots were harvested for this investigation. Separately, the leaves and stem bark were air dried in the shade before being mashed into tiny particles with an electric blender (WESTPOINT M012) as described by Krakowska-Sieprawska et al., 2022 [22]. Extraction of active compounds from leaves and stem bark was conducted in accordance with the method described by Ong Es et al., 2006 [23] with minor modifications as per method described by Zekeya et al.,, 2022 [14] where the leaves and stem bark were then extracted separately in 1000 ml of petroleum ether, dichloromethane, ethyl acetate, and ethanol each, for 48 h twice. The extracts were filtered through muslin cloth on a plug of glass wool in a glass column, and solvents were evaporated in vacuum using a rotary evaporator. Water extracts were prepared by boiling 100 g of stem bark in 1L of water for 10 min and infusing 50 g of leaves in the stem bark decoction. Following that, the concoction was filtered with muslin cloth and lyophilized to yield dry extract. Before further usage, all extracts were refrigerated at 4 °C.

### Determination of bioactive metabolites

The determination of active metabolites from extracts was performed according to the method described by Sinan et al. 2020 and John et al., 2014 [18, 24].

### Determination of phenol

Two ml of Ferric Chloride (FeCl_3)_ solution were added to the 2 ml of 100 mg/ml of each extract and fraction, the appearance of deep bluish-green solution indicated the presence of phenolic compounds.

### Determination of flavonoid

The presence of flavonoid was determined by addition of 5 ml of dilute NH_4_OH into 2 ml of 100 mg/ml of extracts followed by addition of few drops of concentrated Sulphuric acid (H_2_SO_4_). Thereafter, a yellow coloration indicated the presence of flavonoid compounds.

### Test for tannin

To test for tannin, 100 mg of each extract/fraction was boiled in 2 ml of water in a test tube and then filtered, followed by the addition of a few drops of 0.1% FeCl_3_ solution. The presence of tannin compounds was confirmed by the appearance of a brownish green, blue black color.

### Determination of saponin

In a test tube, 100 mg of each extract/fraction was dissolved in 2 ml of distilled water and warmed before being violently shaken. The presence of saponin compounds was indicated by the production of froth lasting at least a minute.

### Determination for antioxidant

One hundred milligram grams (mg) of each sample was dissolved in 1 ml of extractor solvents, filtered, and divided equally between two test tubes. The mixture was agitated and left to stand for 1 min before adding 0.5 ml of pre-prepared 0.1 mM 2,2-diphenyl-1-picrylhydrazyl (DPPH) in one of the test tubes while DPPH was not added to the second test tube which was set as the control. The presence of antioxidant chemicals in the extract was shown by the production of discoloration in comparison to the control.

### LC–MS/MS analysis of water extract of bark stems bark and leaves

The LC–MS/MS analysis of water extract was performed according to method described by John et al. 2014 [24]. The Q-orbitrap-Ultra High Performance (Thermo Fisher Scientific) was used for LC–MS/MS analysis of polar extract as per method described by Tyagi and Agarwal, 2017 and Pucot et al. 2021 [25, 26]. The extract was re-dried using Rotavap under reducing pressure with Nitrogen gas flowing at 15psi at 45 °C, and the Liquid Chromatography was eluted by mobile phases of 0.1% formic acid in water followed by 0.1% formic acid in Acetonitrile. The column conditions were 37 °C and 1.9µ of oven temperature and particle size, respectively. The linked MS was scanned in the 150–2000 m/z range with a resolution of 140,000 and an AGC Target1e6. The maximum IT setting was 200 ms with ionization mode (HESI) collision Energy of 45v.

### Determination of antiviral and cytotoxicity activity

All antiviral screening and cytotoxicity experiments were conducted at Basel University in accordance with the method described by Klimkait et al. 1998 [27 and as per WHO Standard operating procedures (SOPs) for handling biohazardous specimens. Coronavirus SARS-CoV-2 –Delta B1 isolate was donated by Basel University.

All infections with live SARS-CoV-2 were strictly performed in a BSL-3 facility of the Basel University, Department of Biomedicine -Petersplatz in Molecular Virology Laboratory in accordance with the WHO and Federal Government of Switzerland (BAG) Laboratory Biosafety Guidance for working with SARS-CoV-2 with permit #A202850/3. The cells used are HeLa-based cells, which contain an LTR-driven lacZ reporter gene, termed as SX-R5 cells. The virus was subsequently grown in Vero E6 cells maintained at Basel University.

### Antiviral activity

**Fig. 1:**
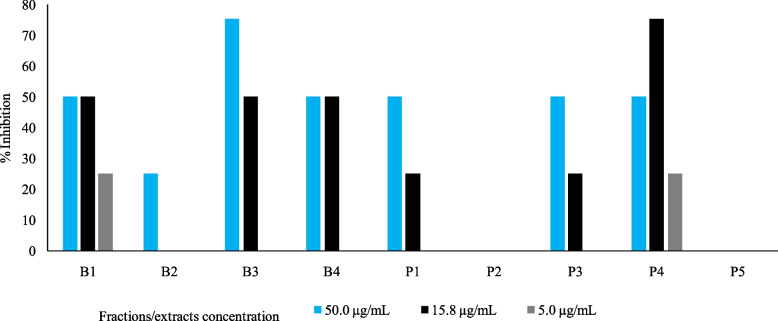
Invitro effect of different concentration of *B. abyssinica* extracts on inhibition of SARS-CoV-2 Delta B1

Antiviral activity was performed according to the method described by Klimkait et al. 1998 [27], with some minor modifications. Extracts/fractions were pre-diluted in a deep-well plate according to the dilution scheme in a way that afterwards the addition of a volume of 50µL would provide the final test concentration on the cells. Remdesivir (RDV) was included as an established and validated activity control. After extract/fraction addition, cultures were transferred to the BSL-3 facility. After about 30 min of preincubation of cells and extract, 100 pfu of the DELTA strain (BS-01) of SARS-CoV-2 virus were added to each culture well. Subsequently, after an adsorption period of 15–30 min, every well was overlayed with low-melting agarose according to the corresponding SOP. Cultures were incubated at 37 °C to allow virus-induced plaques to form. Because cytopathic changes (CPE) develop with time of incubation, the best time of harvest was established by microscopic inspection before the fixative paraformaldehyde (PFA) was added. Quantitative plaque formation was used to demonstrate viral replication in the infectivity range (number of plaques = around 100/well). Plaque reduction at a particular drug concentration was used to assess inhibitory potency. The RDV IC_50_ was at 2.5 µM, which corresponded to the reported activity. The plate on the right is a duplicate plate containing the same component concentrations but no virus. The fixed and stained culture plate is shown on the left plate (Fig. 1). Small white specks were used to represent viral plaques. Compound dilutions were performed from top to bottom, and red lines represent the corresponding compound concentration of the 50% -inhibition of plaque formation (IC_50_).

### Cytotoxicity

**Fig. 2:**
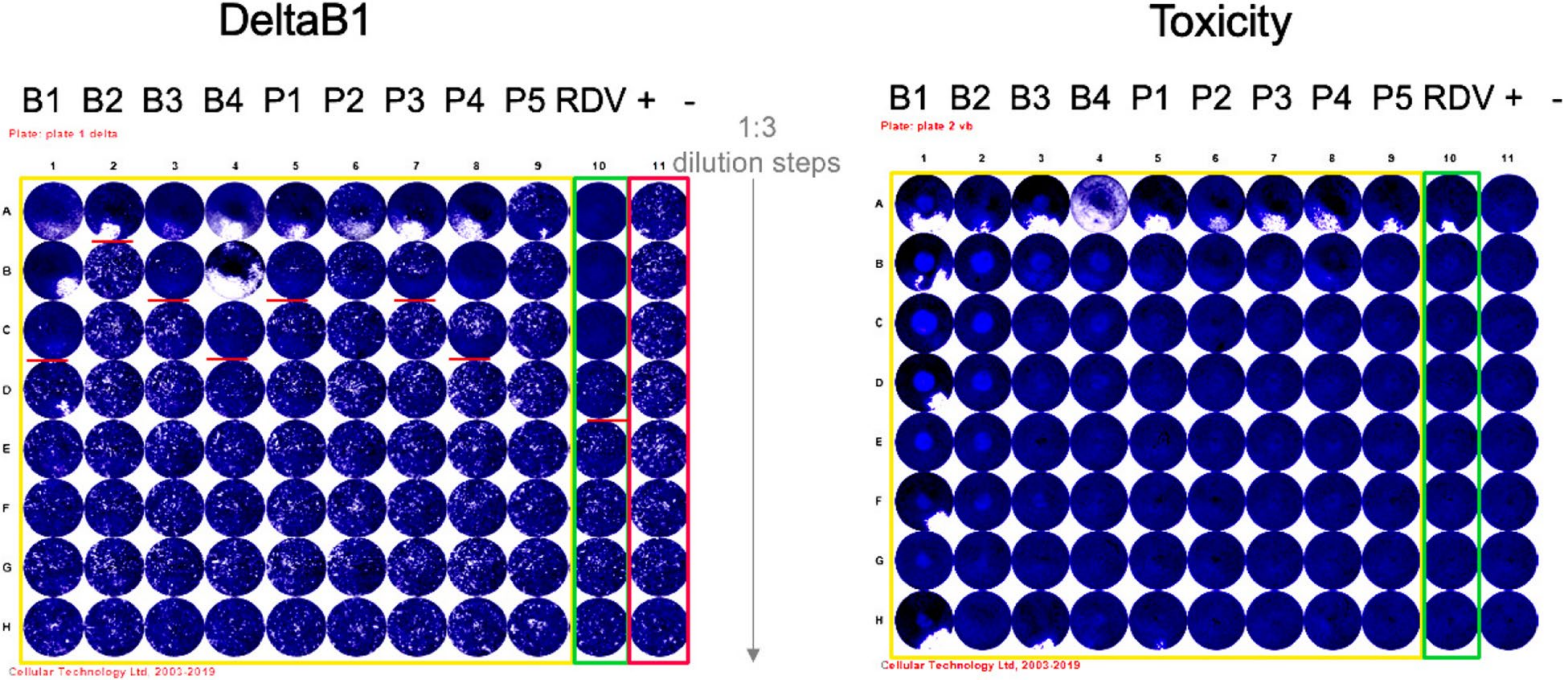
A 96-Well plate showing antiviral assay and cytotoxicity of extracts whereby dilution and antiviral assay (left) and Cytotoxicity assay (right)

The cytotoxicity assay was conducted in accordance with the method described by Klimkait et al. 1998 where all extracts and fractions (named compounds) were pre-diluted in DMSO (analytical grade) (Merck KGaA, Darmstadt, Germany) to obtain stock concentrations of 20 mg/mL. Further dilutions were done in culture medium (DMEM/2%FBS) at ratio of 1:3 until the final extract/fraction concentration as indicated. Cells were pre-seeded on day-1 as detailed in Fig. 1 to allow adherence to the culture plate. Extract/ fraction dilution and dispensing as described by Klimkait et al. 1998 (Fig. 2) to obtain serial dilution of each extract/fraction. This was to cover the entire anticipated biological activity range. DMSO concentrations on the cells were always below 0.5% final concentration to ensure full cell viability. A cell viability plate, using identical extract/fraction concentrations and cell count inhibiting coronavirus delta variant which was included for each extract/fraction as control. The cytotoxicity was also assessed for the extract exposure for 48 h.

## Result

The methanolic and water extracts showed the presence of diverse secondary metabolites (Table 1). However, *B. abyssinica* extracts and fractions possessed high amount of phenolic compounds in both leaves and stem bark fractions of ethyl acetate, methanol and water (Table 1). The results revealed the presence of tannins in stem bark extracts of petroleum ether, dichloromethane, ethanol and methanol whereas non of tannin was revealed in leaves. Flavonoids were also revealed in leaf and stem bark water extracts and fractions of ethyl acetate and methanol. Saponin was shown in petroleum stem bark extract, both leaves and stem bark of methanol and water. However, all fractions and extractsshowed positive antioxidant activity (Table 1).

**Table 1:** Qualitative analysis of selected group of secondary metabolites present in different *B. abyssinica* extracts and fractions

Solvent	Plant part					
		Tannin	Phenol	Flavonoid	Saponin	Antioxidant
Petroleum ether	Stem bark	+	+	-	+	+
	Leaf	-	-	-	-	+
Dichloromethane	Stem bark	+	-	-	-	+
	Leaf	-	-	-	-	+
Ethyl acetate	Stem bark	-	+	+	+	+
	Leaf	-	+	+	+	+
Methanol	Stem bark	+	+	+	-	+
Water	Leaf	+	+	+	+	+
	Stembark + Leaf	+	+	+	+	+

Key: +  = indicates presence of bioactive metabolites and—= indicates absence of bioactive metabolite

The phytochemical analysis of water extracts by LC–MS/MS analysis revealed the presence of active compounds ranging from different phytochemical groups including phenols, coumarin and flavonoids. Compounds namely; 2,4-di-tert-butylphenol, 4-formyl-2-methoxyphenyl propionate 7,8-Dihydroxy-4-methylcoumarin and 2,3, 6-trimethoxyflavone were identified by this study (Table 2).

**Table 2:**
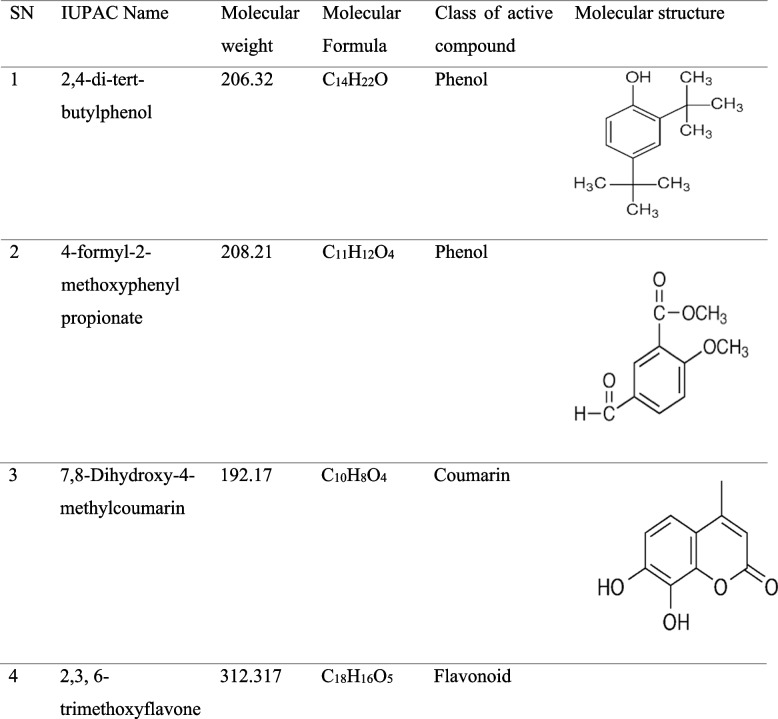
LC-MS/MS Analysis of active compounds of lyophilized water extract of *B. abyssinica* stem bark and leaf

The antiviral activity screening revealed that *B. abyssinica* has active metabolites with a high potential for inhibiting coronavirus, including the Delta variant, which showed higher virulence than its predecessor variants. All fractions demonstrated anti-SARS-CoV-2 (Delta) activity in all concentrations except *B. abyssinica* dichloromethane stembark fraction (P3) and *B. abyssinica* dichloromethane leaf fraction (B2) where the later showed plaque reduction only at the highest concentration of 50 µg/mL, *B. abyssinica* methanolic stembark fraction and *B. abyssinica* methanolic leaf fraction (B1 and P4) exhibited moderate inhibition activity but in all concentrations and the inhibition was dose dependant, the highest dose exhibited high antiviral activity against delta variant.On other hand, P1 and P3 showed plaque reduction at lower dose of 16 µg/mL with exception of *B. abyssinica* petroleum ether stem bark extract (P2) and *B. abyssinica* petroleum ether leaf extract (P5) which did not show any viral inhibition. However, *B. abyssinica* water stem bark and leaf extract (B3) showed high antiviral activity reaching 75% and 50% viral inhibition at Concentration of 50 µg/mL and 16.8 µg/mL and no activity at 5 µg/mL. The activity of extracts and fraction were dose dependant and although most extracts exhibited activity at effective at concentration of 16 µg/mL (Fig. 1).

The water extract (B3) exhibited the highest inhibitory activity against Delta B1 by causing 75% viral death with no cytotoxicity effect on host cells at both 16 µg/mL and 50 µg/mL concentration. The methanolic leaf fraction (P4) also showed activity against SARS CoV -2, where 75% inhibitory activity was observed at concentration of 16 µg/mL and lowered to 50% at 50 µg/mL and to 25% at 5 µg/mL with no cytotoxicity effect on host cells in all. However, the slight cytotoxicity effect on host cells was observed in ethylacetate stem bark fraction (B4) at a concentration of 50 µg/mL (Fig. 2).

## Discussion

Herbs including *Bersama abyssinica* have for long been used to treat infectious diseases in traditional treatment systems in various African countries [14, 18]. This plant has a diverse set of active metabolites, including antioxidants [28], and others with antiviral properties [29]. *B. abyssinica* concoction of stembark and leaves which are key components of Coviba Dawa, herbal preparation in use in Tanzania exhibit a high content of phenolic, tannin and flavonoid compounds in the dry water extract of stem bark and leaves. All extracts have shown remarkable antioxidant activity that could be responsible for viral inhibition as revealed by other studies [30]. Several studies revealed the presence of phenolic compounds which could be effective against viruses including SARS-CoV-2 [31, 32]. The phytochemical screening revealed high amount of phenolics in *B. abyssinica* stem bark and leaves water extracts, which could be associated with high inhibitory activity against SARS-CoV-2 Beta B1 compared to methanolic, ethyl acetate and petroleum ether extract with low phenolic content. The effectiveness of *B. abyssinica* water extract could be due high phenolic compounds that have been revealed by other studies to possess high antiviral activity [33, 34]. The presence of tannins in stem bark extracts seems to have antiviral activity on coronavirus [35, 36] where similar studies revealed similar activity of tannic acid on SARS-CoV-2 [37]. High amount of flavonoids were also revealed leaves extract of *B. abyssinica* which has been reported by several studies to have activity against SARS-CoV-2 [38]. This was revealed by high use of citrus fruits during Covid-19 eruption [39]. Saponin was also revealed in petroleum ether and ethyl acetate extracts of leaves which is also reported to have high inhibitory activity against viruses including SARS CoV-2 due to production of soap-like foaming responsible for antimicrobial activity [40, 41].

Generally, water extract exhibited varied types of active metabolites with two phenolics, coumari and flavonoid which could have synergetic activity against SARS-CoV-2. The increased activity of water extract could be attributed to a high concentration of polar molecules, particularly phenolic compounds with high antioxidant and antiviral properties [42]. Again, the presence of coumarin and flavonoids contributed to antiviral activity which havebeen reported by other studies to have high antiviral activity against SARS-CoV-2 [43, 44].

The presence of 2,4-di-tert-butylphenol in water extract could have contributed to activity against SARS-CoV-2, which is reported by other studies [45, 46] and presence of 7,8-Dihydroxy-4-methylcoumarin and 2,3, 6-trimethoxyflavone in the same extract have increased activity against SARS-CoV-2, which was reported to have antioxidant properties [47, 48], enhancing viral inhibition. In addition, 4-formyl-2-methoxyphenyl propionate was also identified in water extract which has various pharmacological uses including anticardiovasular, antioxidant and anti-inflammatory [49] that would have enhanced inhibition in SARS-CoV-2 B1. Other studies revealed that plants and foods with antioxidants are used for treatment of early stages of COVID-19 [50, 51]. It was also revealed that antioxidant combat viral infection through boosting immune system for protection against SARS-CoV-2 [52]. Recent study in Egypt revealed that medicinal plants have possessed high active metabolites for inhibition of Coronavirus [53]. The results from this study are supported by previous works that revealed the antiviral activity of herbal medicines though ant oxidation, anticoagulation and anti-inhibitory activity of natural phenolic compounds [54–56]. Several efforts toward discovery of SARS Cov-2 have been investigated though preclinical and clinical trials [57]. The findings showed high in vitro antiviral and, the cytotoxicity of *B. abyssinica* extracts and fractions that justifies the use of plant for medicinal purpose and could be potential agent for clinical trials in Tanzania.

## Conclusion

This study discovered the presence of four active compounds in *Bersama abysinica* water extract which possessed the novel antiviral activity against SARS-CoV-2 Delta B1 by 75% viral inhibition with no cytotoxicity effect on cells. The ethylacetate fraction of *B. abyssinica* leaves also showed 50% inhibition of viral activity in vitro, indicating the high potential of these substances as future anti-viral/anti-microbial agents. We therefore recommend isolation of active compounds for further profiling and development with a broader concentration range with twofold dilutions. We further recommend studies that determine the antiviral activity of extracts of all extracts and compounds of *B. abyssinica* on other viral pathogens of clinical concern.

## Supplementary Information

**Additional file 1.**

**Additional file 2.**

## Data Availability

Supplementary files; 1 B. abbyssinica phytochemical bioassay and 2. B. abyssinica antiviral bioassay, are provided with this submission.

## Abbreviations

MUHAS: Muhimbili University of Health and Allied Sciences
IUCN: International Union for Conservation of Nature
SARS-Cov-2: Severe acute respiratory syndrome coronavirus-2
B1: *Bersama abyssinica* Methanolic stembark fraction
P4: *Bersama abyssinica* Methanolic leaf fraction
B2: *Bersama abyssinica* Dichloromethane leaf fraction
P3: *Bersama abyssinica* Dichloromethane stembark fraction t
B3: *Bersama abyssinica* Water stem bark and leaf extract
B4: *Bersama abyssinica* Ethylacetate stembark fraction
P1: *Bersama abyssinica* Ethylacetate leaf fraction
P2: *Bersama abyssinica* Petroleum ether extract
P5: *Bersama abyssinica* Petroleum ether leaf extract
