# Supplementary material for: Screening and evaluation of cytotoxicity and antiviral effects of secondary metabolites from water extracts of Bersama abyssinica against SARS-CoV-2 Delta
Source: BMC Complement Med Ther. 2022 Oct 26;22:280. doi: 10.1186/s12906-022-03754-3 (PMC9598020; doi:10.1186/s12906-022-03754-3)
Supplement: Supplementary file 1 — Additional file 1. [file 12906_2022_3754_MOESM1_ESM.pdf]

Universität Basel
Departement Biomedizin

Departement Biomedizin Basel

Prof. Dr. Thomas Klimkait
University of Basel, Department Biomedicine – Petersplatz
Molecular Virology
Petersplatz 10, CH-4009 Basel
Switzerland

Antiviral Bioassay

All experiments were conducted according to the previously established SOPs; all infections with live SARS-CoV-2 were strictly performed in the BSL-3 facility of the institute in accordance with the official authorization for work with SARS-CoV-2 by the Federal Government of Switzerland (BAG), permit #A202850/3.

Details of the experimental setup:
All compounds were pre-diluted in DMSO (research grade) to obtain stock concentrations of 20mg/mL. Further dilutions were done in culture medium (DMEM/2%FBS) until the final compound concentration as indicated.
Cells were pre-seeded on day -1 as detailed below to allow adherence to the culture plate.

SARS-CoV-2 screening_Tansania-ND

aim: screening compounds with SARS-CoV-2 delta virus

project: Tansania-ND

date 2021-10-18

compounds:

MV-ID	*Sample Code*	*mass (mg)*	*type*	*dissolution*	*stock (mg/mL)*	*DMSO (µL)*	*to test*	*Storage*	
B1	BALET(7)	50	extract	DMSO	20.0	2500	dose response	RT	
B2	BASP(1)	50	extract	DMSO	20.0	2500	dose response	RT	
B3	BASET(3)	50	extract	DMSO	20.0	2500	dose response	RT	
B4	BASET(11)	50	extract	DMSO	20.0	2500	dose response	RT	not in list
P1	P1	5	pure compound	DMSO	20.0	250	dose response	RT	
P2	P2	5	pure compound	DMSO	20.0	250	dose response	RT	
P3	P3	5	pure compound	DMSO	20.0	250	dose response	RT	
P4	P4	10	pure compound	DMSO	20.0	500	dose response	RT	
P5	P5	10	pure compound	DMSO	20.0	500	dose response	RT	

Steps

1	Seed cells.	50 µL	2% FBS
2	Prepare compound dilutions.		in 2% FBS
3	Dispense compounds in screening	50 µL	
4	Add virus, about 100 PFU		1 h adherence
5	Add agarose	100 µL	
6	Incubation	2 days	
7	Readout		

experiment

d 0-1

seed cells

cell type	Vero E6 (Bern)			
medium	DMEM, 2% FBS, 1% PS	volume/well:	50	µL
cells/plate	3.50E+06	total volume / plate	5	mL
cell count / mL	1.71E+06	volume cells/plate:	2.0	mL
viability	70%	volume media/plate:	3.0	mL
passage	7			

Nr of plate:	1	screening	Volume = 50uL / well
Nr of plate:	1	viability	Volume = 50uL / well

incubate o/n @ 37°C

Universität Basel
Departement Biomedizin

Departement Biomedizin Basel

Prof. Dr. Thomas Klimkait
University of Basel, Department Biomedicine – Petersplatz
Molecular Virology
Petersplatz 10, CH-4009 Basel
Switzerland

Compound dilution and dispensing was as described below to obtain serial dilutions of each compound. This was to cover the entire anticipated biological activity range. DMSO concentrations on the cells were always below 0.5% final concentration to ensure full cell viability.
A cell viability plate, using identical compound concentrations and cell count but without viral infection was included for each compound as control.

prepare compound dilutions (96-dwp)

medium: DMEM, 2% FBS, 1% PS

volume compound needed: 50 µL* 6 300 µL / deepwell for inhibition & viability

compounds

										% DMSO
stock :	20	mg/mL	tube							100
Predilution 1: 10	2	mg/mL	tube			1000	µL	100.0	900	15.0
dilution 1: 10	200.0	µg/mL	dwp			800	µL	80.0	720	1.5
				--> serial dilution 1:	3.17	700	µL	220.8	479	
dilution w/ cells 1: 2	100.0	µg/mL	screening			100	µL	50	50	0.8
dilution w/ agarose 1: 2	50.0	µg/mL	plate			200	µL	100	100	0.38

Remdesivir: starting from 50uM

												% DMSO
	stock:	20000	uM	original tube								100
dilution 1:	10	2000	uM	tube			500	µL	50.0	450		15.0
dilution 1:	10	200	uM	dwp			700	µL	70.0	630	5% DMSO	1.5
					--> serial dilution 1:	*3.17*	700	µL	220.8	479.2		
dilution w/ cells 1:	2	100	uM	screening			100	µL	10	90		0.8
dilution w/ agarose 1:	2	50	uM	plate			200	µL	100	100		0.38

dispense 50 µL compounds into 96-wp (2x inhibition, 2x viability)

viability: add 100 µL medium to viability wells/plates

Compounds were pre-diluted in a deep-well plate according to the dilution scheme (top graph below) in a way that afterwards the addition of a volume of 50µL will provide the final test concentration on the cells.
Remdesivir (RDV) was included as established and validated activity control.
After compound addition, cultures were transferred to the BSL-3 facility. After about 30 minutes of preincubation of cells and compound, 100 pfu of the DELTA strain (BS-01) of SARS-CoV-2 virus were added to each culture well.
Subsequently, after an adsorption period of 15-30 minutes, every well was overlayed with low-melting agarose according to the corresponding SOP.
Cultures were incubated at 37°C as described below to allow virus-induced plaques to form.

Prof. Dr. Thomas Klimkait
University of Basel, Department Biomedicine – Petersplatz
Molecular Virology
Petersplatz 10, CH-4009 Basel
Switzerland

deep-wp

compounds	1	2	3	4	5	6	7	8	9	RDV		
	1	2	3	4	5	6	7	8	9	10	11	12
A	200.0	200.0	200.0	200.0	200.0	200.0	200.0	200.0	200.0			
B	63.1	63.1	63.1	63.1	63.1	63.1	63.1	63.1	63.1			
C	19.9	19.9	19.9	19.9	19.9	19.9	19.9	19.9	19.9			
D	6.3	6.3	6.3	6.3	6.3	6.3	6.3	6.3	6.3			
E	2.0	2.0	2.0	2.0	2.0	2.0	2.0	2.0	2.0			
F	0.6	0.6	0.6	0.6	0.6	0.6	0.6	0.6	0.6			
G	0.2	0.2	0.2	0.2	0.2	0.2	0.2	0.2	0.2			
H	0.1	0.1	0.1	0.1	0.1	0.1	0.1	0.1	0.1			

dispense compounds into 96-wp (1x inhibition, 1x viability) 50ul/ well

scr. plate

compounds	1	2	3	4	5	6	7	8	9	RDV		
	1	2	3	4	5	6	7	8	9	10	11	12
A	50.0	15.8	5.0	1.6	0.5	0.2	0.0	0.0	0.0	0.0		
B	50.0	15.8	5.0	1.6	0.5	0.2	0.0	0.0	0.0	0.0		
C	50.0	15.8	5.0	1.6	0.5	0.2	0.0	0.0	0.0	0.0		
D	50.0	15.8	5.0	1.6	0.5	0.2	0.0	0.0	0.0	0.0		
E	50.0	15.8	5.0	1.6	0.5	0.2	0.0	0.0	0.0	0.0		
F	50.0	15.8	5.0	1.6	0.5	0.2	0.0	0.0	0.0	0.0		
G	50.0	15.8	5.0	1.6	0.5	0.2	0.0	0.0	0.0	0.0		
H	50.0	15.8	5.0	1.6	0.5	0.2	0.0	0.0	0.0	0.0		

virus, no substance

cells only

transfer plates to BSL3
add 100 µL medium to viability plates

Virus: add virus to 96-wp ad 10ul

	**Delta B1**	Delta 30.07.2021 MJL
volume:	5	µL / well

total volume 1200 ul
dilution 1: 2
600 ul virus
+ 600 ul media

R02_Box07

adsorbtion time 15 min
prewarm 2x overlay media to 44°C in waterbath
add 100 µL agarose to each well
media used instead of agarose

Incubate at 37 °C for 2-3 days 2 days

After 48 h of incubation at 34°C, cells were processed following the steps as detailed below. As cytopathic changes (CPE) develop within hours, the optimal time of harvest was determined by microscopic inspection, before paraformaldehyde (PFA) was added as fixative:

Prof. Dr. Thomas Klimkait
University of Basel, Department Biomedicine – Petersplatz
Molecular Virology
Petersplatz 10, CH-4009 Basel
Switzerland

Evaluation

d2

> check for CPE under microscope
> add 80 µL 3.7% PFA per well
> incubate for 1 hour
> discard all supernatant
> add 50 µL Crystal Violet per well
> incubate for 5 min
> wash with tap water

Quantitative plaque formation served as proof for viral replication in the infectivity range (no. of plaques = ca. 100 / well). The inhibitory potency was judged as plaque reduction at a given compound concentration. The IC_{50} for the RDV was at ca. 2.5µM, correlating with the reported activity.

The plate on the right is a replicate plate with the same compound concentrations but NO virus added. Slight toxicity was seen for BASET-11 (B4) at a concentration of 50µg/mL. *(The white patches near the 6 o'clock position in some wells are of technical nature during pipetting and can be ignored!).*
→Of note, the cytotoxicity was also only assessed for the compound exposure of 48hrs. This time window may be too short to profile and verify the true cytotoxic influences on eukaryotic cells!

The plate on the left displays the fixed and stained culture plate. Viral plaques are visualized as small white spots. Compound dilutions are from top to bottom, and red lines indicate the respective compound concentration of the 50%-inhibition of plaque formation (IC_{50}):

Prof. Dr. Thomas Klimkait
University of Basel, Department Biomedicine – Petersplatz
Molecular Virology
Petersplatz 10, CH-4009 Basel
Switzerland

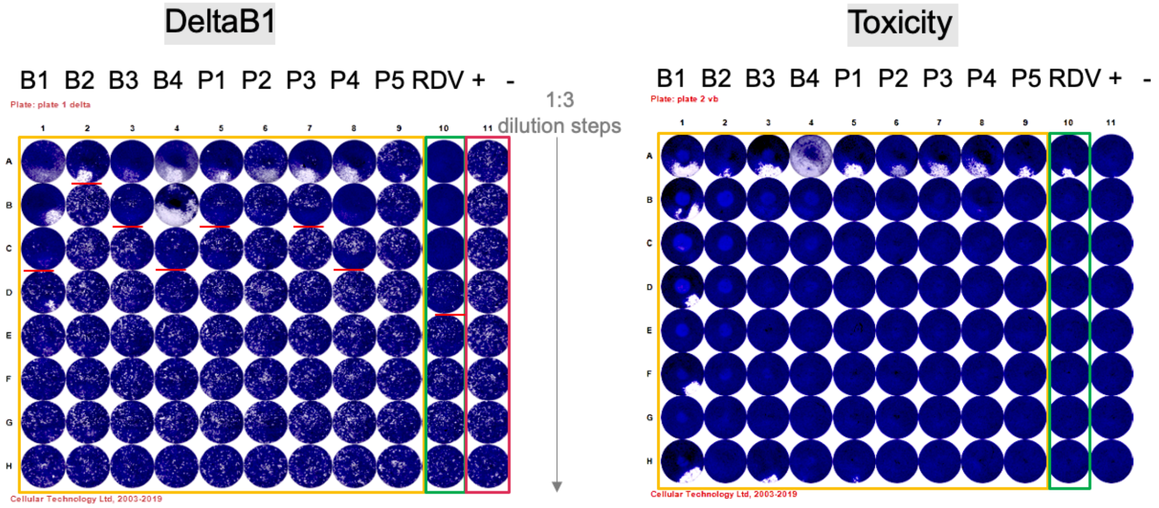

Remdesivir, start at 50µM → 1:3* in serial dilution

Componds, start at 50 µg/ml → 1:3* in serial dilution

Reference wells, virus w/o compound

Compounds:

MV-ID	Sample Code
B1	BALET(7)
B2	BASP(1)
B3	BASET(3)
B4	BASET(11)
P1	P1
P2	P2
P3	P3
P4	P4
P5	P5

**precise dilution factor is: 3.17*

The results of the original plates were then plotted for each compound as semi-quantitative activity (Y-axis) as a function of the compound concentration (X-axis), as shown in the bar graph below.

The provided compounds except P2 and P5 had some anti-SARS-CoV-2(Delta) activity:

- BASP(1)=(B2) only at the highest concentration of 50µg/mL;
- BASET(3)=(B3), P1, and P3 showed a plaque reduction also at 16µg/mL.
- Compounds BALET(7)=B1) and P4 showed a dose-dependent inhibition with significant activity in the absence of cytotoxicity also at 5 µg/mL. Details are displayed below:

Universität Basel
Departement Biomedizin

Departement Biomedizin Basel

Prof. Dr. Thomas Klimkait
University of Basel, Department Biomedicine – Petersplatz
Molecular Virology
Petersplatz 10, CH-4009 Basel
Switzerland

Results

% inhibition

		1	2	3	4	5	6	7	8	9
		BALET(7)	BASP(1)	BASET(3)	BASET(11)	P1	P2	P3	P4	P5
		B1	B2	B3	B4	P1	P2	P3	P4	P5
50.0	µM	*50	25	75	*50	*50	*0	*50	*50	0
15.8	µM	50	0	50	*50	25	0	25	*75	0
5.0	µM	25	0	0	0	0	0	0	25	0
1.6	µM	0	0	0	0	0	0	0	0	0
0.5	µM	0	0	0	0	0	0	0	0	0
0.2	µM	0	0	0	0	0	0	0	0	0

* cytotox, inhibition values imprecise

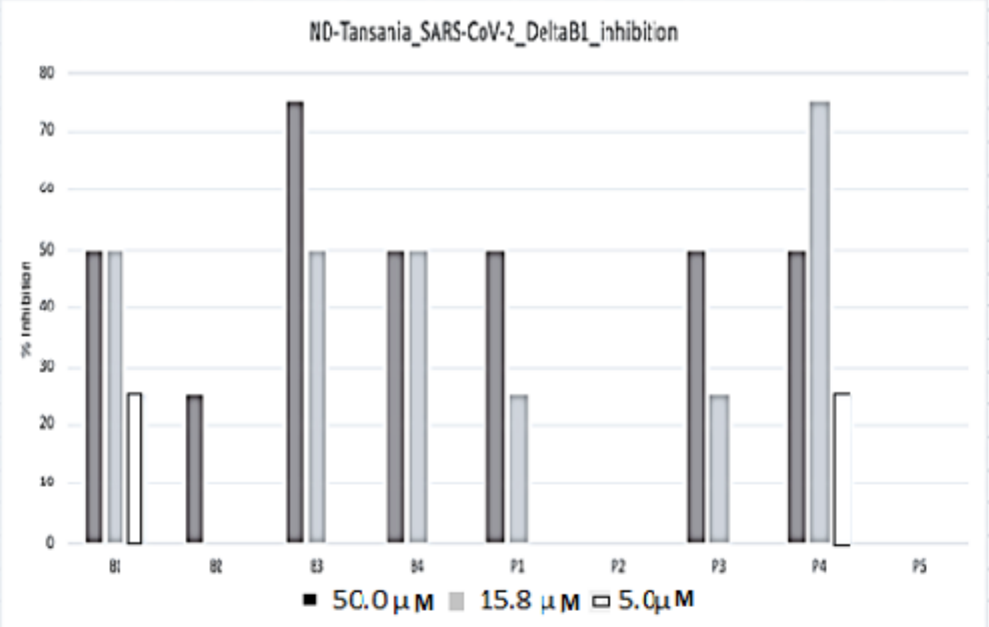

Recommendations:

Should any of the tested compounds be of interest for further profiling and development,

- The respective compound profile should be **repeated with an independent preparation** of the substance for activity verification, and possibly covering a **broader concentration range** with 2-fold dilutions.
- It may be advisable to test also **other viral isolates of clinical concern** such as SARS-CoV-2 Beta and, once available newer emerging viruses, i.e. SARS-CoV-2 Omicron.

- Moreover, a more **detailed cellular toxicity profile** might be needed, including longer compound exposure (5-7 days) and an assessment of the cell replication profile, and possibly the profiling in several cell types.
