# Supplementary material for: Screening and evaluation of cytotoxicity and antiviral effects of secondary metabolites from water extracts of Bersama abyssinica against SARS-CoV-2 Delta
Source: BMC Complement Med Ther. 2022 Oct 26;22:280. doi: 10.1186/s12906-022-03754-3 (PMC9598020; doi:10.1186/s12906-022-03754-3)
Supplement: Supplementary file 2 — Additional file 2. [file 12906_2022_3754_MOESM2_ESM.pdf]

B. abyssinica phytochemical analysis

(a)Test for saponin

i) Stem bark extract

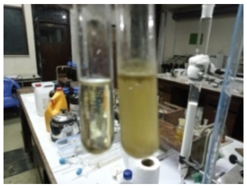 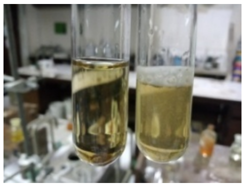 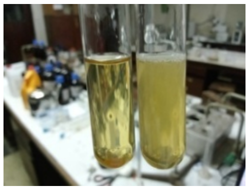 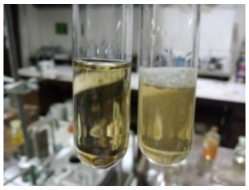 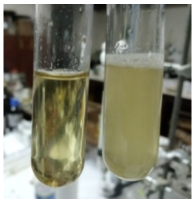

BASP;(X) BASD;(X) BASEA;(X) BASE;(√) BASA;(√)

ii) Leaves extract

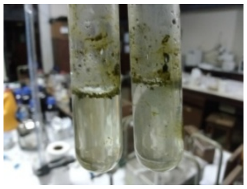 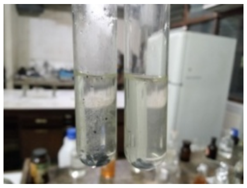 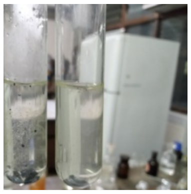 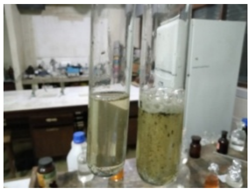 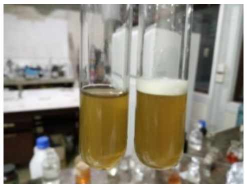

BALP;(X) BALD;(X) BALEA;(X) BALE; (√) BALA;(√)

(b) Test for Tannin

i) Stem bark extract

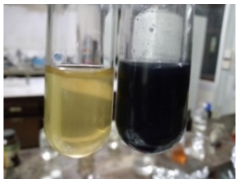 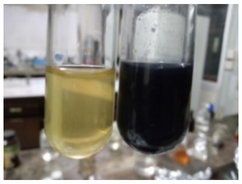 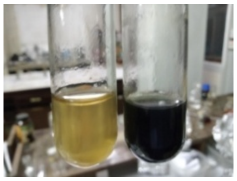 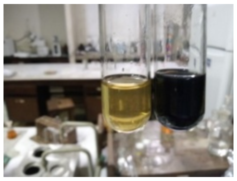 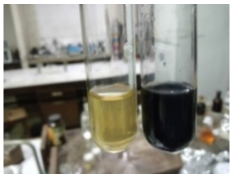

BASP;(√) BASD;(√) BASEA;(√) BASE; (√) BASA;(√)

ii) Leaves extract

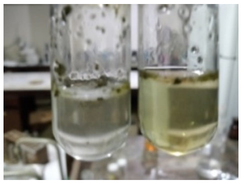 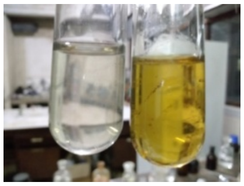 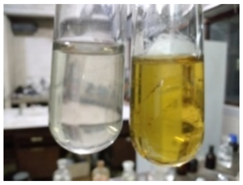 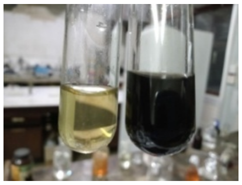 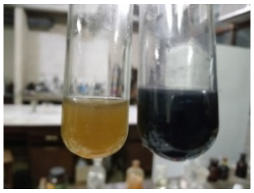

BALP;(X) BALD;(X) BALEA;(X) BALE; (√) BALA;(√)

(c)Test for Flavonoids

i) Stem bark extract

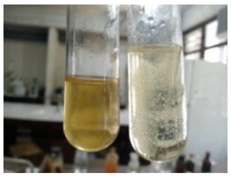 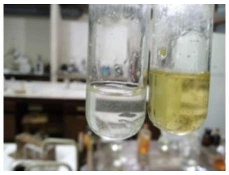 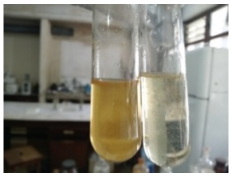 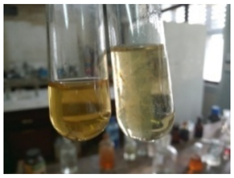 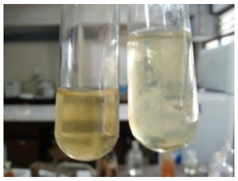

BASP;(X) BASD;(X) BASEA;(X) BASE; (√)BASA; (√)

ii) Leaves extract

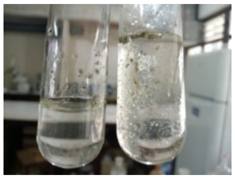 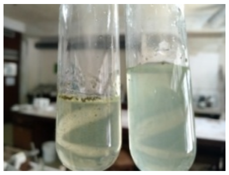 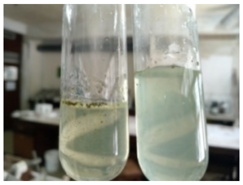 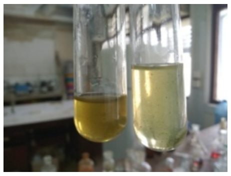 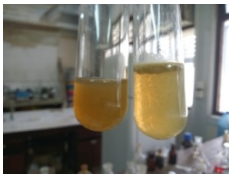

BALP;(X) BALD;(X) BALEA;(X) BALE; (√) BALE;(√)

(d) Test for Phenol

i) Stem bark extract

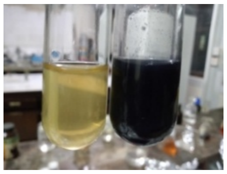 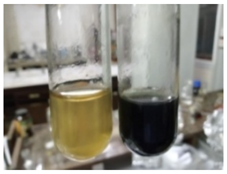 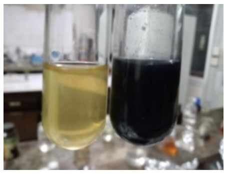 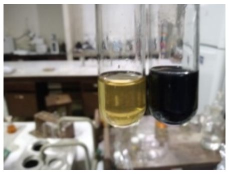 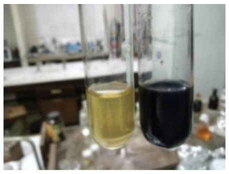

BASP;(√) BASD;(√) BASEA(√) BASE; (√) BASA;(√)

ii) Leaves extract

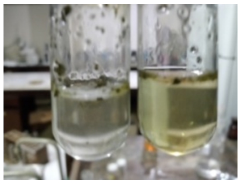 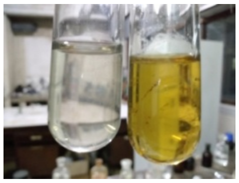 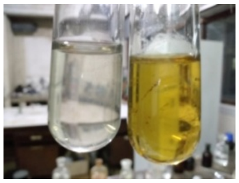 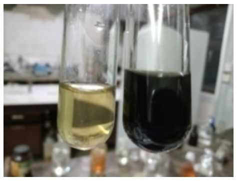 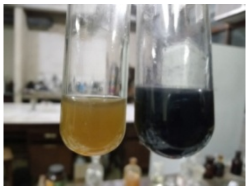

BALP;(X) BALD;(X) BALEA;(X) BALE; (√) BALA;(√)

(e) Test for Antioxidant

i) Stem bark extract

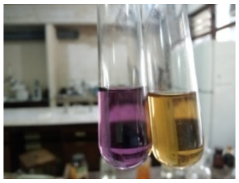
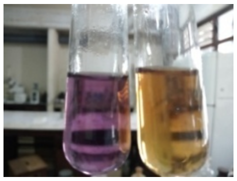
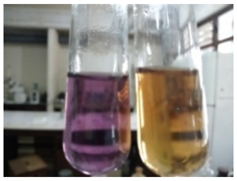
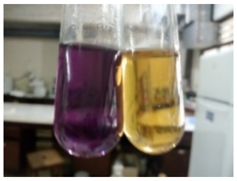
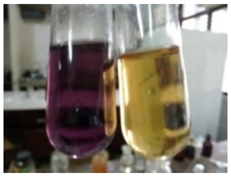

BASP;(√) BASD;(√) BASEA; (√) BASE; (√) BASA;(√)

ii) Leaves extract

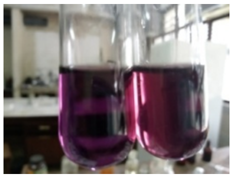
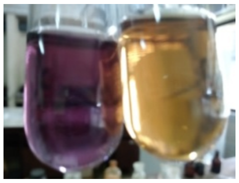
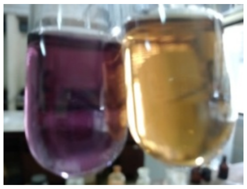
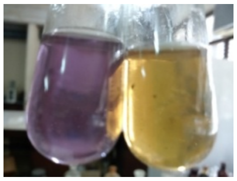
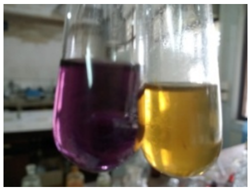

BALP;(X) BALD;(√) BALEA; (√) BALE; (√) BALA;(√)

KEY; B= Bersama, A= Abyssinica, S= Stem, L= Leaves, P=Petroleum ether, D = Dichloromethane,EA=Ethyl acetate, E=Ethanol, A = Aqueous/water, (√) = Present, (X) = Absent
